# Relationship between dialysis quality and brain compliance in patients with end-stage renal disease (ESRD): a cross-sectional study

**DOI:** 10.1590/1516-3180.2021.0117.R1.14092021

**Published:** 2022-04-02

**Authors:** Cristiane Rickli, Danielle Cristyane Kalva, Gustavo Henrique Frigieri, Adriana Fatima Menegat Schuinski, Sérgio Mascarenhas, José Carlos Rebuglio Vellosa

**Affiliations:** I PhD. Professor, Centro Universitário Integrado, Campo Mourão (PR), Brazil.; II PhD. Professor, Biological and Health Sciences Division, Universidade Estadual de Ponta Grossa (UEPG), Ponta Grossa (PR), Brazil.; III PhD. Research Coordinator, Braincare Desenvolvimento e Inovação Tecnológica S.A., São Carlos (SP), Brazil.; IV MD. Professor, Biological and Health Sciences Division, Universidade Estadual de Ponta Grossa (UEPG), Ponta Grossa (PR), Brazil.; V PhD. Founder, Braincare Desenvolvimento e Inovação Tecnológica S.A., São Carlos (SP), Brazil.; VI PhD. Associate Professor, Biological and Health Sciences Division, Universidade Estadual de Ponta Grossa (UEPG), Ponta Grossa (PR), Brazil.

**Keywords:** Renal dialysis, Intracranial pressure, Kidney diseases, Hemodialysis, Chronic kidney disease, Dialysis disequilibrium syndrome, Kt/V

## Abstract

**BACKGROUND:**

The high number of patients with end-stage kidney disease (ESRD) on hemodialysis makes it necessary to conduct studies aimed at improving their quality of life.

**OBJECTIVES:**

To evaluate brain compliance, using the Brain4care method for intracranial pressure (ICP) monitoring, among patients with ESRD before and at the end of the hemodialysis session, and to correlate ICP with the dialysis quality index (Kt/V).

**DESIGN AND SETTING:**

Cross-sectional study conducted at a renal replacement therapy center in Brazil.

**METHODS:**

Sixty volunteers who were undergoing hemodialysis three times a week were included in this study. Brain compliance was assessed before and after hemodialysis using the noninvasive Brain4care method and intracranial pressure wave morphology was analyzed.

**RESULTS:**

Among these 60 ESRD volunteers, 17 (28%) presented altered brain compliance before hemodialysis. After hemodialysis, 12 (20%) exhibited normalization of brain compliance. Moreover, 10 (83%) of the 12 patients whose post-dialysis brain compliance became normalized were seen to present good-quality dialysis, as confirmed by Kt/V > 1.2.

**CONCLUSIONS:**

It can be suggested that changes to cerebral compliance in individuals with ESRD occur frequently and that a good-quality hemodialysis session (Kt/V > 1.2) may be effective for normalizing the patient’s cerebral compliance.

## INTRODUCTION

The number of patients with end-stage kidney disease (ESRD) has been increasing over the years and the treatment method that is most used is hemodialysis (HD).^1,2^ Although the development of HD by Willem Kolff and Belding Scribner has revolutionized the treatment of renal failure, mortality remains significantly high, with high rates of comorbidities and low quality of life.^3^ Several factors influence the risk of mortality among patients undergoing HD,^4^ but high mortality rates may be related to aspects of the dialysis procedure.^5^

To assess the quality of dialysis, the dialysis quality index (Kt/V) is measured, which can be calculated in several ways.^6^ This index needs to be at least 1.2 per dialysis session in order to verify that the session was of good quality. Observational studies have demonstrated a relationship between Kt/V and mortality and morbidity. Patient survival is longer when Kt/V is greater than 1.0.^7-9^

Studies have focused on prevention of and intervention in frequent HD-related complications, given that these complications reduce patients’ quality of life and can contribute to mortality.^10,11^ Approximately 30% of HD sessions have some type of complication,^11^ which may include hypotension, muscle cramps and post-dialysis complaints such as headache, fatigue and inability to concentrate.^12^

Frequent symptoms that are present in dialysis patients, such as headache, nausea and muscle cramps, can represent milder forms of dialysis disequilibrium syndrome (DDS), and this often remains undiagnosed.^13^ DDS occurs due to a sudden drop in urea levels, which results in an osmotic imbalance, with consequent failure of self-regulation of the cerebral circulation, thus leading to cerebral edema. Its clinical presentations depend on the brain region involved: for example, edema in the occipitoparietal subcortical white matter can result in sudden loss of vision and other neurological symptoms.^14^

To prevent this syndrome, it is recommended that high-risk patients should be identified, rapid correction of metabolic acidosis using bicarbonate should be avoided and the urea clearance rate and decrease in plasma osmolality should be controlled.^13^ Considering that cerebral edema and the consequent decrease in cerebral compliance are involved in the pathophysiology of DDS,^15–17^ assessment of intracranial pressure (ICP) could also contribute to a more positive outcome by helping to identify individuals with mild forms of DDS that are hard to recognize.^13^

## OBJECTIVE

The aim of this study was to evaluate the brain compliance of patients with ESRD before and at the end of the hemodialysis session, and to correlate the results obtained with the dialysis quality index (Kt/V).

## METHODS

This was a cross-sectional study at a renal replacement therapy (RRT) center in Brazil. It was conducted after authorization had been received from the Research Ethics Committee of Universidade Estadual de Ponta Grossa (UEPG) (procedural number: 2174527; approved on June 26, 2014).

### Participants

Sixty patients aged 18 years or over, with ESRD, who were undergoing hemodialysis three times a week for 3-4 hours in each session, were included. To define the sample size, studies with similar variables were consulted. However, given that the intracranial pressure variable remains poorly studied, the sample size was defined according to the number of volunteers available. The exclusion criteria were presence of acute infections, chronic viral diseases and pregnancy. All the participants received information about the study and provided written informed consent.

### Clinical and dialysis characteristics

The clinical and dialysis characteristics of the participants were obtained through the computerized system of the RRT center at the Santa Casa de Misericórdia de Ponta Grossa Hospital.

### Assessment of brain compliance

Brain compliance was assessed through the Brain4care method, using equipment provided by Brain4care (São Paulo, SP, Brazil) for non-invasive ICP monitoring. The method is based on measuring volumetric changes in the skull that are detected by a sensor attached to a bandana that is kept in contact with the patient’s head. The equipment filters, amplifies and digitizes the signal coming from the sensor and sends it to a computer. This method has been patented and validated through comparison with the invasive method for monitoring ICP,^18,19^ and it has been registered with the Brazilian National Health Surveillance Agency (ANVISA; registration number 81157910004).

The results are obtained through analysis on the ICP wave pulse morphology. The curve obtained has three peaks: i) P1 is a percussion peak that results from transmission of blood pressure from the choroid plexus; ii) P2 varies according to brain compliance; and iii) P3 is related to closure of the aortic valve in the heart. In situations of intracranial compliance, the amplitudes of the peaks P1, P2 and P3 decrease sequentially.^20,21^ On the other hand, if the cranial adaptive capacity decreases, there is an increase in the ICP, as well as a change in the ICP pulse waveform because the amplitude of the P2 peak becomes higher than those of P1 and P3.^22^

To numerically represent the volunteers’ brain compliance, the ratio between the amplitude of the peaks P1 and P2 was defined as P1/P2 (ratio R = AmpP1/AmpP2). Ideally, the result from this relationship should be > 1.10. A ratio between 1.00 and 1.10 indicates that the patient is on the threshold of abnormality. Values < 1.00 indicate abnormality, i.e. P2 > P1.

Pre-dialysis monitoring was performed before the patient started the hemodialysis session, and the patient was asked to remain immobile for 15-20 minutes of monitoring. At the end of the hemodialysis session, the same procedure was performed.

### Statistical analysis

The Kolmogorov-Smirnov test was used to assess the normality of the data. Since most of the continuous variables did not present normal distribution, these were presented as the median and interquartile range. Categorical variables were presented as absolute numbers (n) and relative frequency (%). The paired parameters obtained pre and post-dialysis were analyzed by means of the Wilcoxon test, and the McNemar test was used to assess the significance of normal and altered ICPs. Possible differences between the groups were showed by means of the chi-square test (χ^2^) for categorical variables and the Mann-Whitney test for continuous variables. The ICP was also evaluated through a classification and regression tree (CART) model using the dialysis quality index (Kt/V) as the dependent variable and the pre and post-dialysis ICPs as independent variables. In all analyses, the significance level was set at P < 0.05. The data were evaluated using the statistical program SPSS 20.0 (SPSS, Chicago, United States).

## RESULTS

The clinical parameters of the patients included in this study are shown in Table 1 and the parameters associated with hemodialysis are shown in Table 2.

**Table 1.** Clinical characteristics of the subjects with end-stage renal disease (ESRD)

Clinical parameters	ESRD (n = 60)
**Age, in years, mean (range)**	60 (50-67)
**Gender, n (%)**	
Male	32 (53)
Female	28 (47)
**Underlying diseases relating to CKD, n (%)**	
Hypertensive nephrosclerosis	35 (59)
Diabetic nephropathy	23 (38)
Polycystic kidney disease	2 (3)
**Length of time on dialysis, in months, mean (range)**	45 (30-68)

Values are expressed as the mean and range or the absolute number (n) and relative frequency (%). CKD = chronic kidney disease.

**Table 2.** Dialysis characteristics of subjects with end-stage renal disease (ESRD)

Dialysis patients’ characteristics	ESRD (n = 60)		
	Pre-dialysis	Post-dialysis	P-value
**Weight (kg)**	70 (62-78)	67 (58-76)	< 0.0001*
**BMI (kg/m^2^)**	26 (22-30)	25 (21-30)	< 0.0001*
**SBP (mmHg)**	155 (128-181)	151 (122-170)	0.212
**DBP (mmHg)**	79 (70-92)	78 (69-88)	0.335
**MBP (mmHg)**	104 (91-123)	103 (88-113)	0.185
**BPM**	76 (67-85)	72 (64-81)	0.050
**Urea (mg/dl)**	102 (88-123)	32 (29-42)	< 0.0001*
**Kt/V**			
> 1.20	-	39 (65)	-
< 1.20	-	21 (35)	-

Values are expressed as the median and interquartile range or absolute number (n) and relative frequency (%); *statistical difference between the groups studied, from Wilcoxon text (P < 0.05); BMI = body mass index; SBP = systolic blood pressure; DBP = diastolic blood pressure; MBP = mean blood pressure; BPM = beats per minute; Kt/V = dialysis quality index.

**Figure 1.**
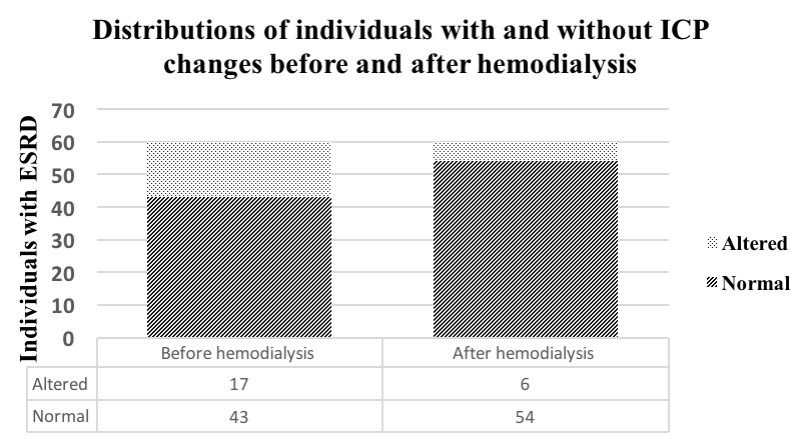
Bar diagram showing the numbers of subjects with ESRD who had normal and altered intracranial pressure before and after hemodialysis.

Figure 1 shows the absolute distributions of individuals with and without ICP changes from before to after hemodialysis. Before the hemodialysis session, 17 individuals (28%) presented ICP changes, while 43 (72%) had normal ICP. After the hemodialysis session, six individuals (10%) were identified as presenting altered ICP and 54 (90%) had normal ICP. Thus, there was a statistical difference (P = 0.035) from before to after hemodialysis for patients with ESRD. It is important to highlight that, out of the 17 patients who presented altered ICP pre-dialysis, 12 presented normal brain compliance after the session, while five continued to present altered ICP. Among the 43 patients who had normal pre-dialysis ICP, one exhibited abnormal ICP after the session.

ESRD = end-stage renal disease; ICP = intracranial pressure.

Table 3 presents a comparison of the clinical and dialysis parameters of the patients with ESRD and their cerebral compliance (normal or altered), to show whether there was any parameter that was related to patients with worse cerebral compliance.

**Table 3.** Comparison of clinical and dialysis variables of subjects with end-stage renal disease (ESRD), according to intracranial pressure: normal or altered

Parameters	ESRD		
	ICP parameters		P-value
	Normal (n = 42)	Altered (n = 18)	
**Clinical**			
**Age, in years**^**a**^	62 (55-67)	50 (44-66)	0.021*
**Gender, n (%)**^**b**^			
Male	25 (60)	7 (39)	0.235
Female	17 (40)	11 (61)	
**Underlying diseases relating to CKD, n (%)**^**c**^			
Hypertensive nephrosclerosis	23 (55)	12 (67)	–
Diabetic nephropathy	17 (40)	6 (33)	–
Polycystic kidney disease	2 (5)	0 (0)	–
Time on dialysis, in months^a^	41 (30-65)	49 (32-84)	0.345
**Dialytic**			
**Weight (kg)**^**a**^			
Pre-dialysis	69 (63-82)	71 (56-77)	0.425
Post-dialysis	66 (60-80)	68 (54-75)	0.429
**BMI (kg/m^2^)^a^**			
Pre-dialysis	25 (23-31)	24 (21-29)	0.302
Post-dialysis	25 (23-30)	24 (20-28)	0.379
**SBP (mmHg)**^**a**^			
Pre-dialysis	157 (135-190)	134 (121-159)	0.031*
Post-dialysis	153 (119-175)	142 (124-154)	0.220
**DBP (mmHg)**^**a**^			
Pre-dialysis	79 (70-91)	79 (67-95)	0.771
Post-dialysis	77 (68-86)	80 (68-91)	0.942
**MBP (mmHg)**^**a**^			
Pre-dialysis	106 (93-128)	103 (85-113)	0.208
Post-dialysis	102 (88-118)	103 (90-110)	0.463
**BPM**^**a**^			
Pre-dialysis	74 (63-84)	82 (74- 92)	0.023*
Post-dialysis	70 (62-77)	79 (71-91)	0.008*
**Urea (mg/dl)**^**a**^			
Pre-dialysis	102 (84-127)	99 (89-116)	0.910
Post-dialysis	37 (28-45)	32 (29-39)	0.375
**Kt/V**^**b**^			
< 1.20	16 (38)	5 (28)	0.637
> 1.20	26 (62)	13 (72)	

Values are expressed as the median and interquartile range or absolute number (n) and relative frequency (%); ^a^Mann-Whitney test; ^b^chi-square χ^2^ test; ^c^descriptive statistics; *statistical difference between the groups studied (P < 0.05); CKD = chronic kidney disease; BMI = body mass index; SBP = systolic blood pressure; DBP = diastolic blood pressure; MBP = mean blood pressure; BPM = beats per minute; Kt/V = dialysis quality index.

**Figure 2.**
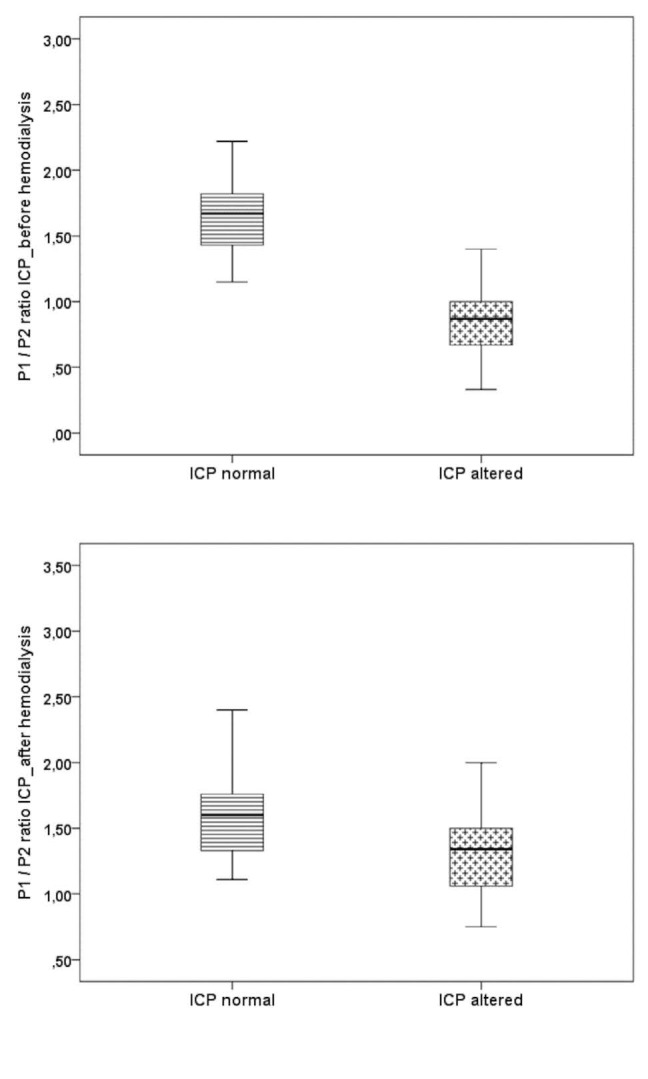
Boxplot showing the median and interquartile range of the P1/P2 ratio for ICP, before and after hemodialysis, among subjects with end-stage renal disease who presented normal and altered intracranial pressure.

A comparison of the P1/P2 ratio of ICP before and after dialysis, for patients with ESRD who presented normal and altered brain compliance, is shown in Figure 2. Individuals with normal brain compliance showed median values for the P1/P2 ratio of 1.67 (range, 1.43-1.83) and those with altered cerebral compliance, 0.87 (range, 0.67-1.00). This difference in the pre-dialysis evaluation was statistically significant (P < 0.001). After dialysis, the patients with normal cerebral compliance exhibited median values of 1.60 (range, 1.33-1.77) and those with altered compliance, 1.34 (range, 1.02-1.50), which was also a statistical difference (P = 0.004). The increase in the P1/P2 ratio after dialysis may indicate an improvement in brain compliance in patients who presented changes in ICP parameters prior to hemodialysis treatment.

ICP = intracranial pressure.

**Figure 3.**
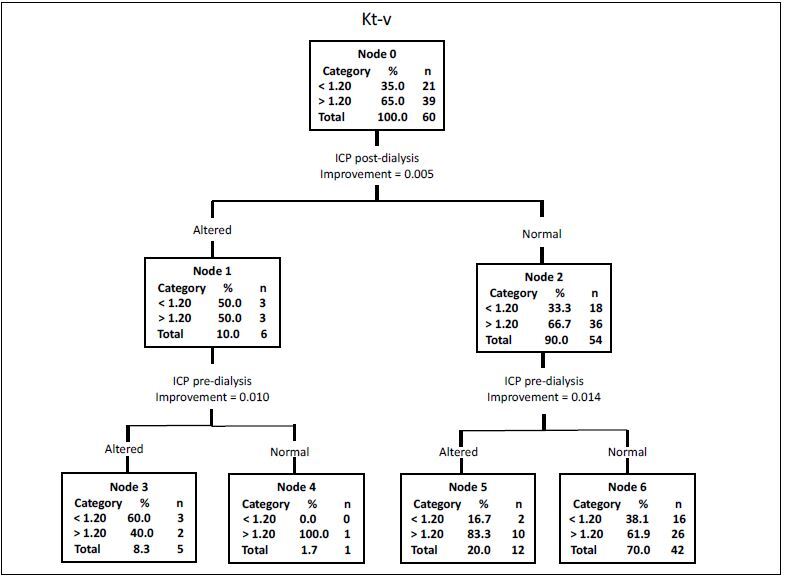
Classification and regression tree (CART) for dialysis quality (Kt/V) in relation to pre and post-dialysis intracranial pressure.

Through a classification and regression tree (CART) (Figure 3), it was visualized that, out of the 60 volunteers with ESRD, 21 (35%) had a Kt/V < 1.20 and 39 (65%) had a Kt/V > 1.20. After the hemodialysis session, there were six individuals with abnormal brain compliance: three with a Kt/V < 1.20 and three > 1.20. Among these six individuals, five of them showed altered brain compliance before the hemodialysis session. One of them presented normal brain compliance before the dialysis and altered compliance afterwards. Also in relation to pre-dialysis brain compliance, out of the 12 individuals whose brain compliance was altered before dialysis and became improved through it, 10 of them had a Kt/V >1.20. Therefore, it can be suggested that efficient dialysis may have helped in improving cerebral compliance.

## DISCUSSION

In the present study, the main finding was that the alteration in pre-dialysis brain compliance that was observed in 28% of the volunteers showed a tendency to become normalized, as seen immediately after the dialysis. Moreover, this study presented the possibility that this result may have been related to HD quality.

Previous studies that assessed the ICP of patients with renal failure did so through correlating the altered results with DDS.^16,23,24^ However, no studies had previously assessed the brain compliance of routine HD patients, because the technique most used is highly invasive.

Noninvasive assessment of cerebral compliance through the Brain4care method could be useful in monitoring patients with ESRD in HD. It would constitute an additional tool for clinical evaluation of these patients and would possibly help in early detection of complications. This method has already been used in several situations such as epilepsy,^25^ hydrocephalus,^26^ cryptococcal meningitis associated with HIV infection,^27^ traumatic brain injury,^19^ hemorrhagic stroke^28^ and assessment of cerebral compliance in the elderly,^29^ among others.

ICP refers to the pressure inside the skull, which is influenced by blood and cerebral parenchyma and by the circulatory dynamics of cerebrospinal fluid (CSF). If there is an increase in the proportion of one of these components (blood, fluid or parenchyma) and the cerebral adaptive capacity is exceeded, the ICP increases.^30,31^

The aim of HD is to simulate the process of glomerular ultrafiltration. It is based on the principle of diffusion, in which clearance or removal of a high concentration of uremic toxins present in the blood is achieved by means of migrating the blood through a semipermeable membrane (the dialyzer or filter), to form a solution of lower concentration, called the dialyzed solution.^32^

A normal ICP waveform (Figure 3) was observed in 90% of the patients in the present study after dialysis. Considering the mechanisms of HD and the pathophysiology of changes to ICP, it can be suggested that the removal of fluids that occurs in HD directly influences the maintenance of the balance between blood, CSF and cerebral parenchyma, thereby normalizing ICP.

According to Robertson et al.,^33^ patients with intracranial hypertension often have elevated blood pressure due to sympathetic hyperactivity, especially in cases of head trauma. However, in the present study, patients with altered ICP parameters had lower systolic blood pressure (SBP). At the same time, it was found that patients with changes to their cerebral compliance had higher heart rate values, both pre and post-dialysis. Dimitri et al.^34^ suggested that there was direct interaction or communication between the heart and the brain, with the observation that when the ICP rose, the heart rate also increased. This is important information, because it can assist in validation of the ICP parameters for assessing cerebral compliance.

Another important result from the present study was the relationship between cerebral compliance and the best-quality dialysis, as shown by Kt/V > 1.20. It can be suggested that good-quality dialysis can help to improve the post-dialysis ICP parameters of patients with ESRD who presented altered brain compliance before HD.

In this study, one volunteer showed changes to his brain compliance only after dialysis. No complications were reported in this patient’s medical records during his HD session, and his Kt/V was 1.25. In addition, it was not possible to establish any relationship between this finding and the patient’s clinical and dialysis parameters.

In the formula of the Kt/V ratio, K refers to the dialyzer urea clearance, which is multiplied by the treatment time (t) and divided by the patient’s urea distribution volume (V). K depends on the blood flow rate, size of the dialyzer and flow of the dialysate, t varies from three to four hours and the urea distribution volume of the patient (V) corresponds to approximately 55% of the individual’s body weight.^35^ This volume can be more accurately estimated through an anthropometric equation that uses an individual’s gender, age, height and weight (e.g. the Watson equation).^36^ Based on the above, several factors can influence the quality of HD and be reflected in the Kt/V ratio, such as the duration of the session and the interdialytic weight gain, which needs to be controlled by the patient through dietary care.

Inadequate hemodialysis (Kt/V < 1.20) can occur due to low adherence or non-adherence to treatment recommendations (such as fluid restriction, regular frequency of dialysis sessions and adherence to 240-minute sessions) and because of the clearance limitations of the conventional HD technique. It has been shown that not attending just one dialysis session is associated with a 25%-30% increase in the risk of death. This is a common problem with regard to HD.^12^

According to Kimata et al.,^37^ small changes in the way of conducting HD sessions, such as increasing the blood flow rate to 200 ml/min and the treatment time to four hours for some patients, can decrease the percentage of patients with Kt/V < 1.20, with a consequent increase in survival among these patients.

However, four-hour dialysis sessions do not guarantee good-quality dialysis. It is necessary to consider the residual renal function, the Kt/V ratio normalized according to body surface area and the expected ultrafiltration rate. In addition, it is important to consider the patients’ acceptance of the duration of dialysis to which they are subjected, since this directly impacts their quality of life and treatment adherence.^38^

Madero and Sarnak^39^ questioned whether the hemodialysis procedure might be partly responsible for brain structural and cognitive changes, such as cerebral edema, which leads to increased ICP. However, our results suggested that an adequate hemodialysis session, with Kt/V > 1.20, may be effective in normalizing the ICP of individuals who have presented changes.

Lastly, it is worth mentioning that the clinical assessment is superior to any Kt/V formula and should serve as the basis for determining the adequacy of dialysis.^6^ Thus, the importance of clinical assessment of cerebral compliance among patients undergoing HD can be emphasized.

## CONCLUSION

Through noninvasive assessment of the ICP parameters by means of the Brain4care^®^ method, it could be seen that changes to cerebral compliance among patients on hemodialysis can occur frequently. Moreover, it can be suggested that good-quality hemodialysis (i.e. when Kt/V is greater than 1.20) may help to normalize the ICP parameters. Thus, the importance of maintaining a Kt/V ratio of at least 1.20 can be emphasized, in order to ensure good-quality dialysis for patients with ESRD.
